# Can routine laboratory tests discriminate SARS‐CoV‐2‐infected pneumonia from other causes of community‐acquired pneumonia?

**DOI:** 10.1002/ctm2.23

**Published:** 2020-06-05

**Authors:** Yunbao Pan, Guangming Ye, Xiantao Zeng, Guohong Liu, Xiaojiao Zeng, Xianghu Jiang, Jin Zhao, Liangjun Chen, Shuang Guo, Qiaoling Deng, Xiaoyue Hong, Ying Yang, Yirong Li, Xinghuan Wang

**Affiliations:** ^1^ Department of Laboratory Medicine Zhongnan Hospital of Wuhan University Wuhan University Wuhan China; ^2^ Center for Evidence‐Based and Translational Medicine Zhongnan Hospital of Wuhan University Wuhan China; ^3^ Department of Radiology Zhongnan Hospital of Wuhan University Wuhan University Wuhan China

**Keywords:** 2019 novel coronavirus, community‐acquired pneumonia, COVID‐19, routine laboratory tests, SARS‐CoV‐2

## Abstract

**Background:**

The clinical presentation of SARS‐CoV‐2‐infected pneumonia (COVID‐19) resembles that of other etiologies of community‐acquired pneumonia (CAP). We aimed to identify clinical laboratory features to distinguish COVID‐19 from CAP.

**Methods:**

We compared the hematological and biochemical features of 84 patients with COVID‐19 at hospital admission and 221 patients with CAP. Parameters independently predictive of COVID‐19 were calculated by multivariate logistic regression. The receiver operating characteristic (ROC) curves were generated and the area under the ROC curve (AUC) was measured to evaluate the discriminative ability.

**Results:**

Most hematological and biochemical indexes of patients with COVID‐19 were significantly different from patients with CAP. Nine laboratory parameters were identified to be predictive of a diagnosis of COVID‐19. The AUCs demonstrated good discriminatory ability for red cell distribution width (RDW) with an AUC of 0.87 and hemoglobin with an AUC of 0.81. Red blood cell, albumin, eosinophil, hematocrit, alkaline phosphatase, and mean platelet volume had fair discriminatory ability. Combinations of any two parameters performed better than did the RDW alone.

**Conclusions:**

Routine laboratory examinations may be helpful for the diagnosis of COVID‐19. Application of laboratory tests may help to optimize the use of isolation rooms for patients when they present with unexplained febrile respiratory illnesses.

## 1 INTRODUCTION

Chinese people are facing unprecedented panic induced by the outbreak of 2019 novel coronavirus (SARS‐CoV‐2, also known as 2019‐nCov) infected pneumonia (COVID‐19) in Wuhan, China since December 2019.^1^ Currently, the SARS‐CoV‐2 spread quickly among other cities and countries. As of March 21, 2020, approximately 292 142 confirmed cases have been reported worldwide.^2^

The SARS‐CoV‐2 is considered a relative of the deadly Middle East respiratory syndrome (MERS) and severe acute respiratory syndrome (SARS) coronaviruses. The COVID‐19 patients are characterized by pneumonia symptoms, such as fever, radiographic evidence of pneumonia, respiratory symptoms, and possibly transmitted from animal to human.^3^, ^4^, ^5^ The public health authorities proposed COVID‐19 definitions that combined clinical features (e.g., fever, cough, and anhelation) with epidemiological factors (e.g., travel to a seafood and wet animal wholesale market in Wuhan or direct contact with another patient with COVID‐19) to improve diagnostic accuracy.^6^, ^7^ Unfortunately, these epidemiological features are not specific and have poor positive predictive value during the outbreak.

The COVID‐19 appears to cause symptoms similar to other etiologies of community‐acquired pneumonia (CAP) based on clinical data from 41 COVID‐19 patients,^3^ and can spread from humans to humans.^3^, ^8^ Distinguishing COVID‐19 from other etiologies of CAP is one of the major challenges of the COVID‐19 outbreak. Despite recommendations that examining hematological and biochemical parameters as part of the diagnostic workup for COVID‐19,^3^, ^9^ it is urgent to evaluate the ability of these features to accurately discriminate cases of COVID‐19 from cases of CAP. Thus, we conducted the current study aiming to evaluate the ability of routine laboratory tests for distinguishing COVID‐19 from other etiologies of CAP and help health workers to effectively, quickly, and calmly deal with COVID‐19.

## 2 MATERIALS AND METHODS

### 2.1 Patients and data collection

To determine the ability of routine laboratory tests to differentiate COVID‐19 from CAP due to other causes, we compared the hematological and biochemical data of COVID‐19 and CAP patients. The 84 COVID‐19 patients presented to our hospital from December 26, 2019 to January 30, 2020. The patients were laboratory confirmed SARS‐CoV‐2 infection by real‐time RT‐PCR. The CAP group consisted of 221 patients who visited our hospital from January 2018 to December 2018. All of the medical records were reviewed retrospectively. The inclusion criteria of CAP group consisted of (a) patients had ≥2 symptoms and signs of CAP and had evidence of pneumonia revealed by the emergency department physician or internal medicine consultant, (b) patients with a complete record of hematological and biochemical indicators, and (c) hospitalized patients. Exclusion criteria were as follows: (a) patients deficient in clinical hematological and biochemical data and (b) outpatient. Patients were diagnosed according to the World Health Organization interim guidance for COVID‐19 and divided into mild and severe groups. Healthy controls included 120 healthy people who made the physical check‐up in our hospital from December 13, 2019 to December 17, 2019. The clinical data collection from patients was approved by the Ethics Committee of Zhongnan Hospital of Wuhan University. Written informed consent was waived by the Ethics Commission for emerging infectious diseases.

### 2.2 Hematological and serum biochemical examination

Routine hematological and serum biochemical examination was ordered at the discretion of the physicians and performed using standard methods in our hospital. Routine peripheral blood cells, including hemoglobin (HGB), lymphocytes, and monocytes, were analyzed. Routine serum biochemical parameters, including alanine aminotransferase (ALT), aspartate aminotransferase (AST), AST/ALT ratio, total bilirubin, direct bilirubin, unconjugated bilirubin, total protein (TP), albumin (ALB), globulin (GLB), γ‐glutamyl transpeptidase (GGT), alkaline phosphatase (ALP), and total bile acid (TBA) were measured.

### 2.3 Statistical analysis

Statistical analyses were conducted using IBM SPSS version 22.0 software. Statistical analysis for the results was performed using the Student's *t*‐test for only two groups or using one‐way analysis of variance for more than two groups. The receiver operating characteristic (ROC) curves were generated, and the area under the ROC curve (AUC) was measured to evaluate the discriminative ability.^10^ Higher AUC was considered to show better discriminatory ability as follows: excellent, AUC of ≥0.90; good, 0.80 ≤ AUC < 0.90; and fair, 0.70 ≤ AUC < 0.80. A *P*‐value of <0.05 was considered statistically significant.

## 3 RESULTS

**FIGURE 1:**
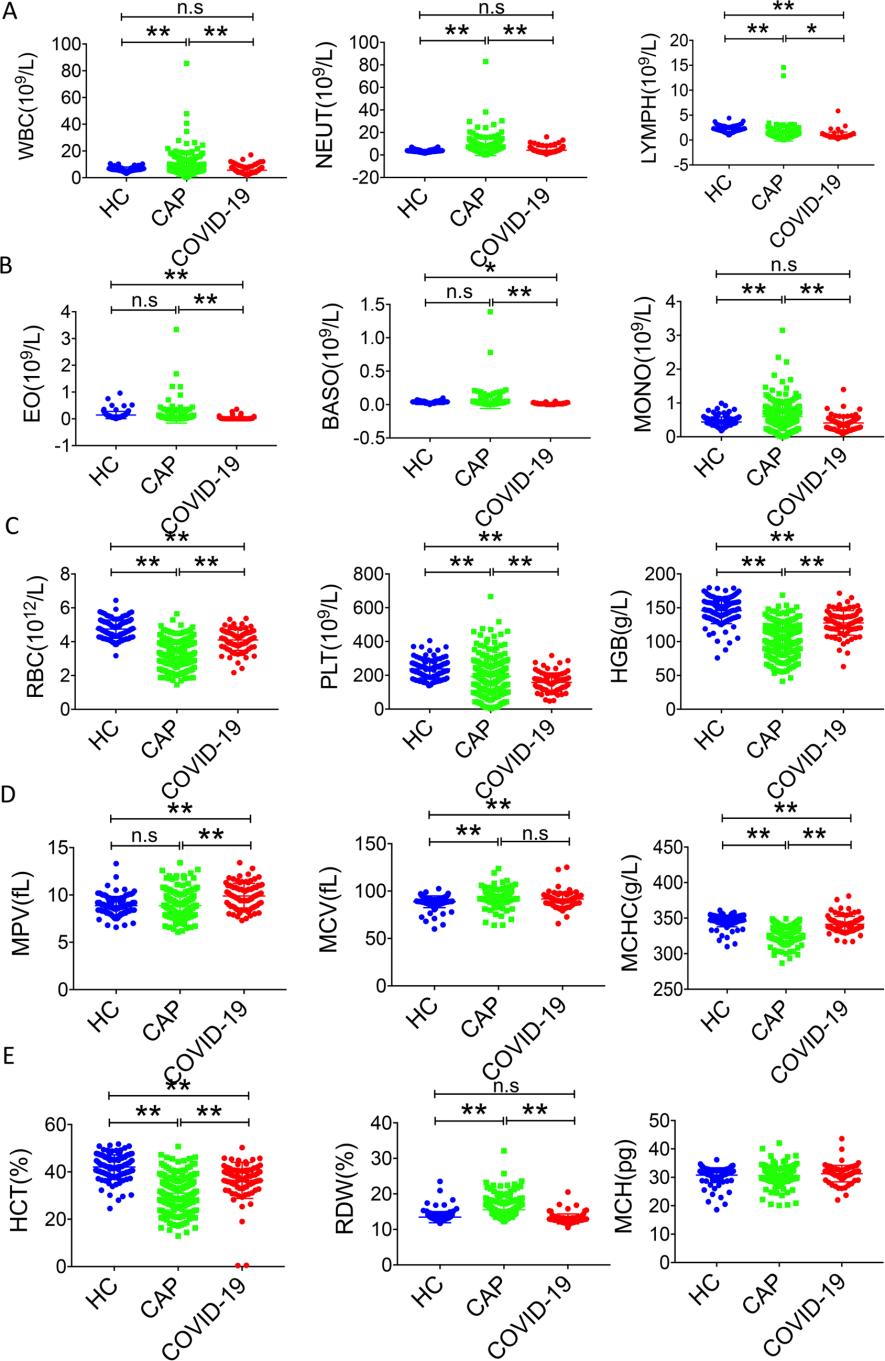
General characteristics of hematological parameters among healthy control (HC), CAP patients, and COVID‐19 patients. *P < .05, **P < .01, n.s, not significant

We have retrieved data of 221 CAP and 84 COVID‐19 patients with complete hematological and biochemical data used for comparison. Among the 221 CAP patients, 8.1% had viral pneumonia, 81.9% had bacterial pneumonia, 8.1% had fungal pneumonia, and 1.8% had pneumocystosis or chlamydia pneumonia. Eighty‐four COVID‐19 and 219 CAP patients received PCT examination; 71 COVID‐19 and 142 CAP patients received CRP test. The mean values of laboratory indexes at the time of hospital admission in COVID‐19 and CAP patients are demonstrated in Table 1 and Figure 1. Both COVID‐19 and CAP patients had lower mean lymphocyte and platelet counts than healthy control. COVID‐19 patients had significantly lower mean values for white blood cell (WBC), neutrophil, eosinophil (EO), lymphocyte, monocyte, red cell distribution width (RDW), platelet counts, PCT, and ALB than did patients with CAP. COVID‐19 patients had significantly higher mean values for HGB, hematocrit (HCT), ALB, and total protein (TP) than did CAP patients. There were no significant differences in mean values of ALT, AST, erythrocyte mean levels of GLB, GGT, TBA, CRP, and ALP between COVID‐19 and CAP patients.

**TABLE 1:** Laboratory values at the time of admission to hospital for COVID‐2019 patients and CAP patients

Variable	Healthy control (n = 120)	CAP (n = 221)	COVID‐2019 (n = 84)	*P*
Age (years)	33 (24–39)	71 (56–86)	58 (48–70)	.000
Gender (M/F)	68/52	142/79	51/33	.385
WBC	6.46 (5.56–7.42)	9.01 (4.89–10.27)	5.61 (3.59–7.11)	.000
NEU	3.7 (2.89–4.46)	7.02 (3.2–8.11)	4.25 (2.34–5.11)	.000
EO	0.14 (0.06–0.17)	0.13 (0.01–0.15)	0.02 (0–0.01)	.000
BASO	0.04 (0.03–0.05)	0.05 (0.02–0.06)	0.01 (0.01–0.02)	.001
LYMPH	2.14 (1.8–2.42)	1.2 (0.56–1.48)	0.91 (0.61–1.04)	.000
RBC	4.74 (4.32–5.15)	3.34 (2.83–3.89)	4.1 (3.77–4.54)	.000
HGB	145.7 (134.2–160.1)	102 (85.9–119.3)	127.6 (118.9–138.1)	.000
PLT	237.6 (194.75–268)	193.4 (116–256)	159 (117–189)	.000
MONO	0.44 (0.34–0.51)	0.6 (0.35–0.77)	0.41 (0.26–0.50)	.000
HCT	42.07 (39.05–45.72)	31.01 (26.3–36)	36.4 (33.6–40.7)	.000
MCV	88.81 (87.1–92.1)	93.2 (89.9–96.7)	91.7 (89.1–94.3)	.000
MCH	30.76 (30.1–32.23)	30.6 (29.5–32)	31.3 (30.2–32.4)	.195
MCHC	345.9 (343.2–350.5)	328.6 (324.6–334.5)	341 (334–346)	.000
RDW	13.46 (12.6–13.6)	15.55 (13.7–16.7)	13.0 (12.2–13.2)	.000
MPV	8.86 (8.3–9.4)	8.89 (8–9.5)	9.9 (8.7–10.9)	.000
PCT	na	3 (0.04–0.61)	0.67 (0.02–0.14)	.026
CRP	na	63.7 (9.9–72.1)	76.4 (24.8–102.8)	.305
ALB	na	29.9 (26.6–33.4)	35.4 (32.3–39.1)	.000
TP	na	59.03 (52.7–65.2)	63.5 (60.4–67.5)	.000
ALT	na	30.78 (12–35)	53.9 (18.8–44.3)	.212
AST	na	47.33 (17–39)	64.9 (26.8–69.3)	.249
GLB	na	29.14 (24.7–33)	28.0 (25.3–30.1)	.181
GGT	na	59.62 (19–60)	52 (20–62)	.426
TBA	na	8.09 (2.3–6.7)	5.3 (2.3–6.4)	.050
ALP	na	126.9 (71–120)	80.0 (54.5–84.5)	.142

*Note*: Date are mean value (interquartile range).

Abbreviations: ALB, albumin; ALP, alkaline phosphatase; ALT, alanine aminotransferase; AST, aspartate aminotransferase; BASO, basophile; CRP, C‐reactive protein; DBIL, direct bilirubin; EO, eosinophil; GGT, γ‐glutamyl transpeptidase; GLB, globulin; HCT, hematocrit; HGB, hemoglobin; LYM, lymphocyte; MCH, erythrocyte mean corpuscular hemoglobin; MCHC, erythrocyte mean corpuscular hemoglobin concentrate; MCV, mean corpuscular volume; MONO, monocyte; MPV, mean platelet volume; na, not applicable; NEU, neutrophil; PCT, procalcitonin; PLT, platelet; RBC, red blood cell; RDW, red cell distribution width; TBA, total bile acid; TBIL, total bilirubin; TP, total protein; UBIL, unconjugated bilirubin; WBC, white blood cell.

**FIGURE 2:**
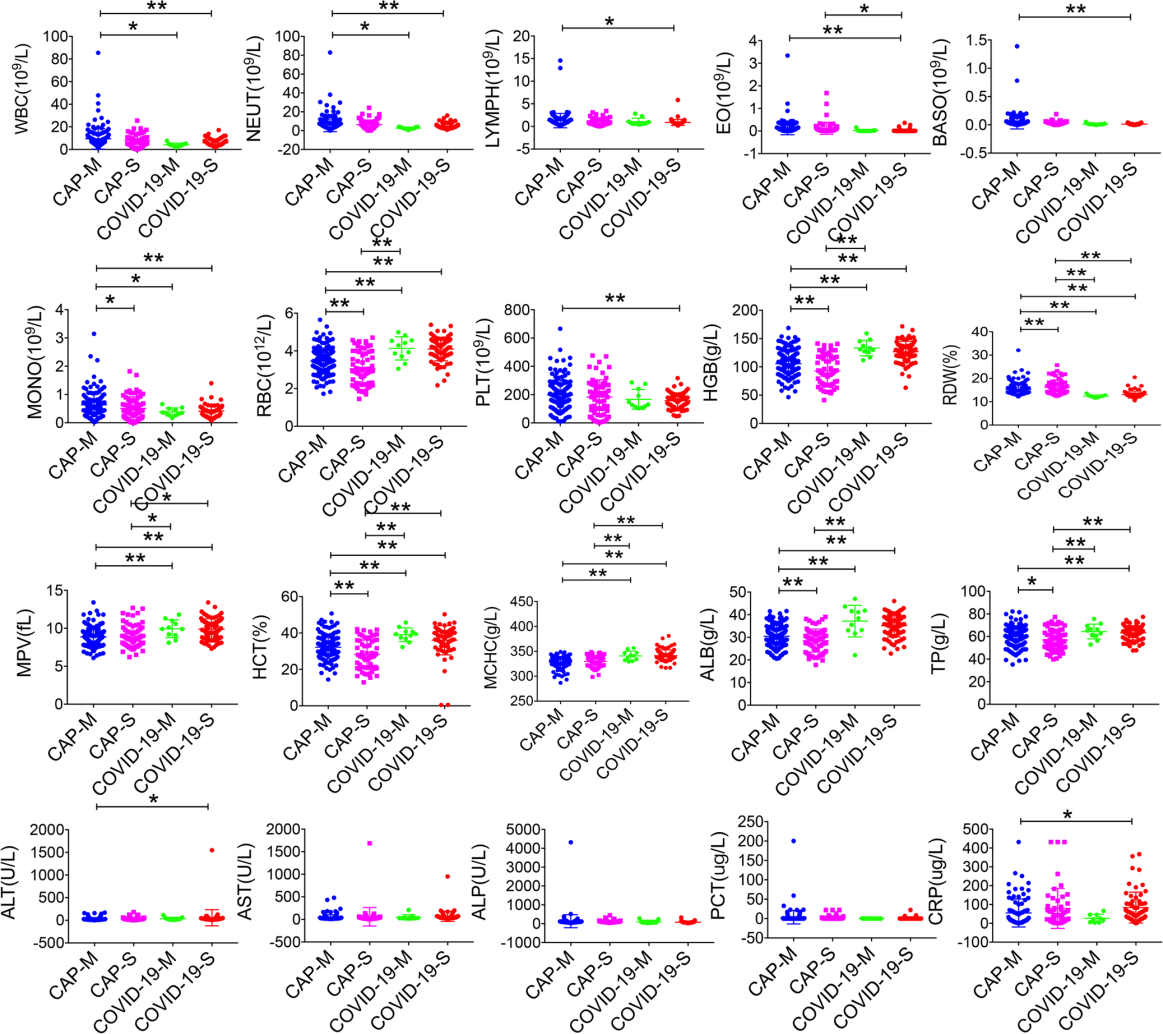
General characteristics of hematological and biochemical parameters among CAP mild patients (CAP‐M), CAP severe patients (CAP‐S), COVID‐19 mild patients (COVID‐19‐M), and COVID‐19 severe patients (COVID‐19‐S). *P < .05, **P < .01

Among the mild group (155 CAP and 11 COVID‐19), CAP patients showed increased RDW, WBC, neutrophil, monocyte, and decreased red blood cell (RBC), HGB, RDW, HCT, ALB, erythrocyte mean corpuscular hemoglobin concentrate, and mean platelet volume (MPV), compared with that of COVID‐19 patients. Similarly, among the severe patients (66 CAP and 73 COVID‐19), CAP patients showed increased RDW, EO and decreased RBC, HGB, HCT, MCHC, MPV, TP, and ALB compared with that of COVID‐19 patients (Figure 2).

The proportion of patients with abnormal laboratory features is presented in Table 2. Both COVID‐19 (81.0%) and CAP (56.6%) patients had lymphopenia. A significantly higher proportion of COVID‐19 patients presented reduced WBC and EO, normal basophile, increased AST and CRP, and normal PCT, whereas a significantly higher proportion of CAP patients had a decrease of RBC, HGB, HCT, ALB, and increase of neutrophil, monocyte, and RDW.

**TABLE 2:** Abnormal laboratory results for COVID‐2019 and CAP patients

Variable	COVID‐2019 (n = 84)	CAP (n = 221)	*P*
WBC			.00
Normal	55 (65.5%)	118 (53.4%)	
Low	20 (23.8%)	27 (12.2%)	
High	9 (10.7%)	76 (34.4%)	
RBC			.00
Normal	51 (60.7%)	36 (16.3%)	
Low	33 (39.3%)	185 (83.7%)	
HGB			.00
Normal	55 (65.5%)	36 (16.2%)	
Low	29 (34.5%)	185 (83.7%)	
PLT			.01
Normal	59 (70.2%)	135 (61.1%)	
Low	25 (29.8%)	65 (29.4%)	
High	0 (0%)	21 (9.5%)	
NEU			.00
Normal	62 (73.8%)	104 (47.1%)	
Low	9 (10.7%)	25 (11.3%)	
High	13 (15.5%)	92 (41.6%)	
LYM			.00
Normal	15 (17.9%)	93 (42.1%)	
Low	68 (81.0%)	125 (56.6%)	
High	1 (1.2%)	3 (1.4%)	
MONO			.00
Normal	73 (86.9%)	111 (50.2%)	
Low	1 (1.2%)	13 (5.9%)	
High	10 (11.9%)	97 (43.9%)	
EO			.00
Normal	19 (22.6%)	145 (65.6%)	
Low	65 (77.4%)	70 (31.7%)	
High	0 (0%)	6 (2.7%)	
BASO			.00
Normal	84 (100%)	177 (80.1%)	
High	0 (0%)	44 (19.9%)	
HCT			.00
Normal	46 (54.8%)	31 (14.0%)	
Low	38 (45.2%)	190 (86.0%)	
MCV			.34
Normal	75 (89.3%)	188 (85.1%)	
Low	5 (6.0%)	11 (5.0%)	
High	4 (4.8%)	22 (10.0%)	
MCH			.96
Normal	72 (85.7%)	192 (86.9%)	
Low	6 (7.1%)	15 (6.8%)	
High	6 (7.1%)	14 (6.3%)	
MCHC			.00
Normal	73 (86.9%)	197 (89.1%)	
Low	0 (0%)	24 (10.9%)	
High	11 (13.1%)	0 (0)	
RDW			.00
Normal	81 (96.4%)	156 (70.6%)	
High	3 (3.6%)	65 (29.4%)	
MPV			.36
Normal	81 (96.4%)	217 (98.2%)	
High	3 (3.6%)	4 (1.8%)	
ALT			.28
Normal	65 (77.4%)	183 (82.8%)	
High	19 (22.6%)	38 (17.2%)	
AST			.00
Normal	52 (61.9%)	178 (80.5%)	
High	32 (38.1%)	43 (19.5%)	
TBIL			.02
Normal	81 (96.4%)	194 (87.8%)	
High	3 (3.6%)	27 (12.2%)	
DBIL			.89
Normal	72 (85.7%)	188 (85.1%)	
High	12 (14.3%)	33 (14.9%)	
UBIL			.02
Normal	82 (97.6%)	195 (88.2%)	
High	2 (2.4%)	23 (10.4%)	
TP			.00
Normal	65 (77.4%)	106 (48.0%)	
Low	19 (22.6%)	115 (52.0%)	
ALB			.00
Normal	50 (59.5%)	36 (16.3%)	
Low	34 (40.5%)	185 (83.7%)	
GLB			.00
Normal	62 (73.8%)	109 (49.3%)	
Low	1 (1.2%)	20 (9.0%)	
High	21 (25%)	92 (41.6%)	
GGT			.83
Normal	59 (70.2%)	158 (71.5%)	
High	25 (29.8%)	63 (28.5%)	
ALP			.01
Normal	77 (91.7%)	175 (79.2%)	
High	7 (8.3%)	46 (20.8%)	
TBA			.37
Normal	80 (95.2%)	204 (92.3%)	
High	4 (4.8%)	17 (7.7%)	
PCT			.00
Normal	44 (52.4%)	55 (24.9%)	
High	40 (47.6%)	164 (74.2%)	
CRP			.00
Normal	7 (8.3%)	38 (17.2%)	
High	64 (76.2%)	104 (47.1%)	

**FIGURE 3:**
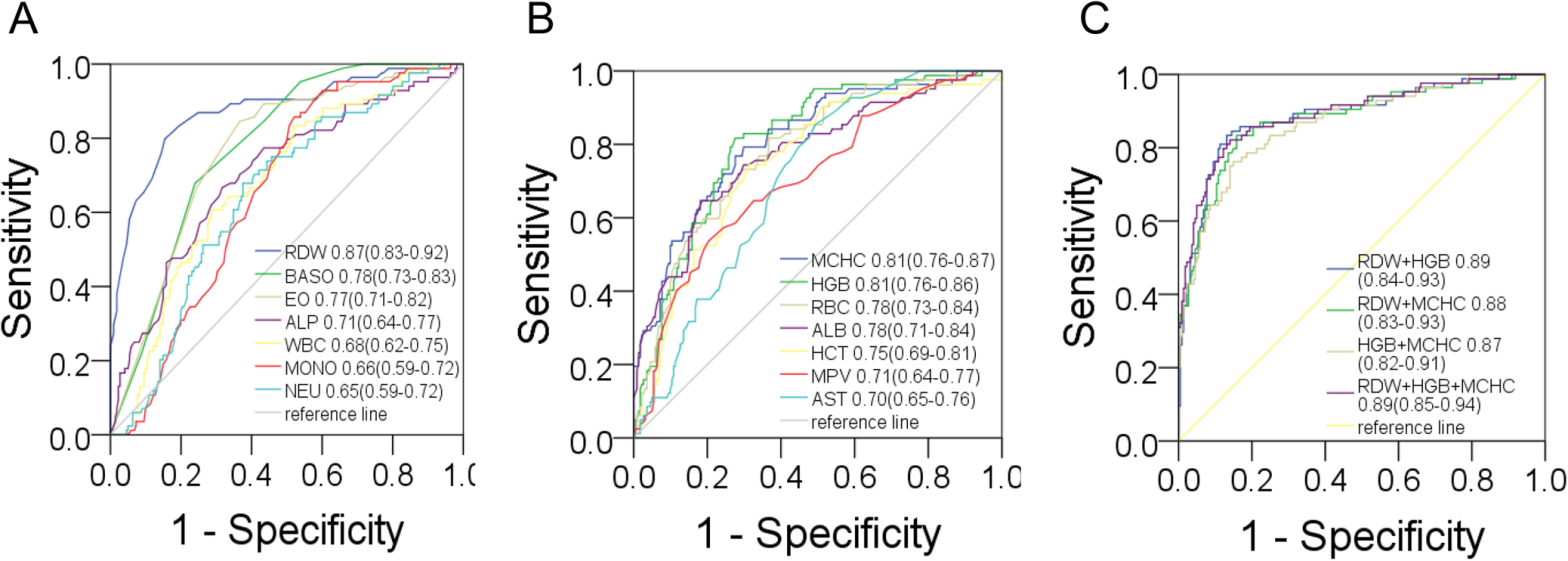
Receiver operator characteristic curves for nine parameters (A), and another six parameters (B), and combinations of the parameters (C), comparing data for CAP patients with that for COVID‐19 patients

Multivariate analysis demonstrated the laboratory indexes independently discriminating COVID‐19 from CAP. The OR of the factors to predict COVID‐19 versus CAP is shown in Table 3. The ROC curves and AUC (Figure 3) demonstrated that RDW, MCHC, and HGB had good discriminatory ability. RBC, ALB, EO, HCT, ALP, and MPV had fair discriminatory ability. Furthermore, the cutoff value for RDW could be 13.35 that provided a reasonable sensitivity (79.8%) or specificity (84.6%). When ROC curves were calculated for combinations of these three parameters, improvement in AUC was presented, with the maximum AUC being 0.89.

**TABLE 3:** Multivariate predictors of COVID‐2019 versus CAP

Variable	OR (95% CI)	*P*
WBC		
Normal		
Low	0.24 (0.05–1.23)	.09
High	2.96 (0.54–16.22)	.21
RBC		
Normal		
Low	5.36 (2.22–12.94)	.00
HGB		
Normal		
Low	5.92 (0.85–41.18)	.07
NEU		
Normal		
Low	4.14 (1.00–17.14)	.05
High	8.81 (3.01–25.81)	.00
LYM		
Normal		
Low	0.27 (0.10–0.76)	.01
High	0.21(0.01–5.74)	.36
EO		
Normal		
Low	0.21 (0.09–0.53)	.00
High	7.24E + 06 (0–)	.99
RDW		
Normal		
High	5.16 (1.33–20.01)	.02
AST		
Normal		
High	0.21 (0.08–0.57)	.00
TP		
Normal		
Low	5.20 (1.93–14.00)	.00
GLB		.01
Normal		
Low	10.47 (0.91–120.75)	.06
High	4.03 (1.48–10.94)	.01
ALP		
Normal		
High	3.88 (1.10–13.64)	.03

OR, odds ratio.

## 4 DISCUSSION

This study indicates that several hematological and biochemical abnormalities occur more frequently in CAP patients than in COVID‐19 patients. The statistically significant difference in mean values was noted for most laboratory features tested. However, to be useful in diagnosis, the overlap in the distribution of the results for patients with COVID‐19 and those with CAP must be small.

SARS‐CoV‐2 spread quickly in China since the first official announcement in December 2019 by the Wuhan Municipal Health Commission.^11^, ^12^ Possibly, the Spring Festival travel rush that millions of people were on the way home and SARS‐CoV‐2 would spread fast especially along with people coming out from Wuhan. Because the confirmation diagnosis of COVID‐19 now mainly relies on PCR assays for the detection of SARS‐CoV‐2,^13^ initial medical examination of potential COVID‐19 depends primarily on clinical, radiographic, and epidemiological features.^9^ Physicians and public health workers keep struggling with the difficult task of evaluating patients for COVID‐19 presenting with unknown febrile respiratory illnesses. As a result, public health authorities went on revising COVID‐19 definitions to improve the accuracy of diagnosis.

Our findings demonstrated that nine laboratory features independently predictive of discernibility between COVID‐19 and CAP. The identification of the RDW, HGB, and MCHC as the best discriminatory ability. The discriminatory ability likely results from elevated HGB and MCHC while low RDW seen in COVID‐19. Our study also highlighted laboratory parameters that are common in both COVID‐19 and CAP, and therefore not useful in differentiating the two diseases. Lymphocytopenia is characteristic and of similar magnitude for both COVID‐19 and CAP. However, lymphocytopenia in COVID‐19 was also accompanied by depletion of EO and normal basophile, whereas it was accompanied by reduced RBC, elevated neutrophil count, and monocyte count in CAP.

Because the CAP cohort did not have laboratory indexes over time, trends in laboratory values were unable to perform after hospital admission. Therefore, more significant differences in the laboratory indexes might occur later in the illness between COVID‐19 and CAP patients. Our findings indicate that simple laboratory tests may help to distinguish COVID‐19 from CAP. Application of these tests together with epidemiological data may be helpful to avoid misdiagnosis of COVID‐19 as CAP and shorten the time of isolation of patients with respiratory symptoms.

## CONFLICT OF INTEREST

The authors declare that there is no conflict of interest.

## Data Availability

The data that support the findings of this study are available from the corresponding author upon reasonable request.
